# Fetal Zika Virus Infection in Vietnam

**DOI:** 10.1371/currents.outbreaks.1c8f631e0ef8cd7777d639eba48647fa

**Published:** 2017-09-05

**Authors:** Phan Trong Lan, Luong Chan Quang, Vu Thi Que Huong, Nguyen Vu Thuong, Phan Cong Hung, Tran Thi Luu Nguyen Huong, Huynh Phuong Thao, Nguyen Thi Thanh Thao, Anthony W Mounts, Leisha D Nolen

**Affiliations:** Director, Pasteur Institute, Ho Chi Minh City, Vietnam; Department of Prevention and Control Diseases, Pasteur Institute, Ho Chi Minh City, Vietnam; Microbiology and Immumnology, Pasteur Institute, Ho Chi Minh City, Vietnam; Deputy Director, Pasteur Institute, Ho Chi Minh City, Vietnam; Department of Prevention and Control Diseases, Pasteur Institute, Ho Chi Minh City, Vietnam; Department of Planning, Pasteur Institute, Ho Chi Minh City, Vietnam; Department of Prevention and Control Diseases, Pasteur Institute, Ho Chi Minh City, Vietnam; Division of Global Health Protection, Centers for Disease Control and Prevention, Atlanta, Georgia, USA; Arctic Investigation Program, Centers for Disease Control and Prevention, Anchorage, Alaska, USA

## Abstract

As of 13 July 2016, 13 countries have reported fetal Zika virus (ZIKV) infection. Here we report a case of fetal ZIKV infection that resulted from an infection originating in Vietnam.

## Report

On March 30, 2016, a woman at eight weeks of pregnancy presented with one day of rash, conjunctivitis, and fatigue to a hospital in Ho Chi Minh City, Vietnam. Blood samples were collected and tested for ZIKV using primers and probes as previously described^1^ . Real-time RT-PCR was positive for ZIKV and negative for rubella virus. Two days after the initial blood draw, a repeat blood sample and a urine sample were collected. The urine sample tested positive for ZIKV by RT-PCR, but the later blood sample was negative. Viral RNA isolated from the urine sample was amplified using previously defined primers ZIKVENVF and ZIKVENVR and sequenced^2^ . Sequence results indicate that the strain isolated from this patient is most closely related to those isolated previously in Malaysia (2012) and French Polynesia (2013).

The patient reported no recent travel. Her husband had been working in Malaysia, returning to visit Vietnam from March 16th to 19th. He reported no symptoms in the month prior to his visit to Vietnam, and no cases of Zika had been reported in the area where he worked. The patient denied any sexual contact 12 days prior to the onset of symptoms, the assumed longest incubation period for Zika^5^. The patient’s two-year old daughter experienced similar symptoms three days before her mother, however samples were only collected after the resolution of symptoms and were negative by RT-PCR.

Seven days after her onset of symptoms the patient was seen in a prenatal clinic for counseling regarding ZIKV infection during pregnancy. A 8-week ultrasound performed the week before had shown a viable fetus, however at the visit, a repeat ultrasound was performed that indicated fetal demise. The following day a suction aspiration abortion was performed, during which samples were taken from both the placenta and fetus. Both the fetal and placental samples were positive by ZIKV RT-PCR, while negative for rubella.

The previous lack of reported microcephaly in Asia and Africa had led to speculation regarding ZIKV strain differences in relation to teratogenicity. This case indicates that the viral strain present in Asia also has the ability to cause infection in utero. It is possible fetal infection was not previously noted in Asia due to limited surveillance systems for birth defects, or to early in life infection that provides immunity at child bearing age, or possibly, due to a more gradual spread of the disease.

This report is one of the first documented cases of ZIKV infection in Vietnam, however it is likely that infection has been occurring for many years without detection or diagnosis. Since the increased awareness of the risk of ZIKV infection, three tourists have been identified who were infected while traveling in Veitnam and two local cases have been confirmed^3^. Based on this report it is important for countries in Southeast Asia to increase surveillance, vector control, and public health messaging and for the global public health system to consider increasing travelers’ awareness of infection risks not only in the Americas, but also in Asia.

## Competing Interests Statement

The authors have declared that no competing interests exist. The authors have no potential bioethics/dual use concerns to disclose.

## Data Availability Statement

There are no additional data to report.

## Corresponding Author

Leisha Nolen, email address: xdf8@cdc.gov.
